# Value of Systematic and MRI‐Ultrasound Fusion Prostate Biopsy in Different Prostate Specific Antigen (PSA) Levels

**DOI:** 10.1002/cnr2.70099

**Published:** 2025-01-21

**Authors:** Solmaz Ohadian Moghadam, Mohammad Haddadi, Erfan Amini, Seyed Ali Momeni, Masoud Bitaraf, Mohammad Reza Nowroozi

**Affiliations:** ^1^ Uro‐Oncology Research Center Tehran University of Medical Sciences Tehran Iran

**Keywords:** fusion biopsy, MRI‐targeted biopsy, prostate cancer, prostate specific antigen, PSA, transrectal biopsy

## Abstract

**Background:**

Current approach to clinically suspicious biopsy‐naïve men consists performing prostate MRI, followed by combined systematic (TRUS‐Bx) and MRI‐Ultrasound fusion biopsy (MRI‐TBx) in those with PIRADS score ≥ 3. Researchers have attempted to determine who benefits from each biopsy method, but the results do not support the safe use of one method alone. This study aims to determine the optimal approach in biopsy‐naïve men, according to their PSA levels.

**Methods and Results:**

A retrospective chart review of clinically suspicious biopsy‐naïve men who underwent both TRUS‐Bx and MRI‐TBx was done. Prostate specific antigen (PSA) levels were compared between patients only positive for MRI‐TBx and those with positive TRUS‐Bx. Further, cancer cases were divided to < 10 and ≥ 10 PSA groups and the pathology results, obtained by each method, were compared. Out of 195 men, 36 were diagnosed with prostate cancer (PCa). PCa was diagnosed by both MRI‐TBx and TRUS‐Bx in 26 men, half of whom had PSA > 10 ng/mL. At PSA ≤ 10 ng/mL, PCa would have been missed in 4 men (11.1%) had MRI‐TBx not been done, and in 6 men (16.6%) had TRUS‐Bx not been done.

**Conclusion:**

Despite attempts to perform only one biopsy method in men with clinical suspicion of prostate cancer, we propose that at least in men with PSA ≤ 10 ng/mL, both systematic and MRI‐targeted biopsies be performed.

## 1 Introduction

Prostate cancer (PCa) is the second most frequent cancer diagnosis and the second leading cause of cancer death among men. Its incidence and burden are increasing universally while its mortality rate is decreasing in developed countries [1, 2]. The latest version of European urology association and its associates (EAU‐EANM‐ESTRO‐ESUR‐ISUP‐SIOG) guideline on prostate cancer indicates that biopsy‐naïve clinically suspicious men, based on abnormal digital rectal examination (DRE) or elevated prostate‐specific antigen (PSA), should undergo magnetic resonance imaging (MRI) before prostate biopsy. Combined targeted and systematic biopsy should be performed in patients with positive MRI, meaning Prostate Imaging Reporting and Data System (PI‐RADS) ≥ 3 [3].

The standard transrectal ultrasonography‐guided biopsy (TRUS‐Bx) of prostate, in which 10 to 12 cores are randomly sampled, is accompanied by the risk of not detecting clinically‐significant prostate cancers (csPCa) due to its random nature, and yet over‐detection of clinically‐insignificant cancers. These lead to inappropriate treatments, avoidable side effects, and unnecessary costs on patients and health systems [4, 5]. Multi‐paramteric (mp) MRI was then extensively utilized in clinical practice and a standardized reporting system, named PI‐RADS, was introduced to avoid variation in interpretation. Later, several studies indicated a higher efficacy for MRI‐targeted biopsy (MRI‐TBx) in detecting csPCa [5, 6, 7]. However, a significant number of csPCa cases (9%–15%) were missed when performing MRI‐TBx alone [7, 8]. These raised the question whether we can use either of the TRUS‐Bx or MRI‐TBx alone (and if yes under which circumstances?), or we are obligated to perform both.

Dell'Oglio et al. tried to develop a pre‐biopsy risk calculator using MRI and clinical/para‐clinical features to spare cases with low risk of harboring csPCa outside the index lesion from TRUS‐Bx. Their results failed to recommend safe use of MRI‐TBx alone [9]. We assessed the association between the biopsy results and PSA levels in a group of biopsy‐naïve men who underwent both methods, in an effort to understand who benefits from each method according to their PSA levels. This study presents the comparison between the results of each biopsy method and PSA levels and proposes suggestions on performing biopsy in biopsy‐naïve men.

## 2 Methods

The Uro‐oncology research center, Tehran, Iran registry database was used to conduct this cross sectional study. Records of all patients visited between January 20, 2021 and March 20, 2022 at urology clinic of Imam Khomeini hospital complex (IKHC), affiliated to Tehran University of Medical Sciences, who had PSA ≥ 3 ng/mL or abnormal DRE with no previous history of PCa were reviewed. This study is approved by research ethics committee of IKHC (IR.TUMS.IKHC.REC.1399.528) and all the methods were carried out in accordance with the declaration of Helsinki. Informed consent was obtained from all patients regarding their procedure and possible use of data for research purposes.

All men with high PSA (≥ 3 ng per milliliter), or abnormal DRE, underwent mpMRI at IKHC using a Siemens 1.5 Magnetom Tesla MRI machine. All images were read by an expert radiologist with experience with prostate MRI. PI‐RADS version 2 was used to score prostate lesions. In patients with multiple lesions, the highest score was considered. PI‐RADS scores range from 1 to 5 and clinical suspicion rises with higher scores. Scores 3–5 indicate combined targeted and systematic biopsy.

Men with PI‐RADS 3–5 and no contraindication underwent MRI‐TBx (2 cores of suspicious lesions) and systematic sampling (10–12 cores) of the prostate done by an expert urologist. Rectal cleansing was done before sampling using povidone‐iodine, and prophylactic antibiotic was administered.

Blood samples were analyzed in central laboratory of IKHC for total PSA. All prostate specimens were assessed by one expert pathologist and Gleason score was reported for each sample. The highest Gleason scores were recorded in the database for systematic and targeted samples, separately. The highest between the two was considered the final score. A score of 7 or higher is considered clinically significant.

PSA levels were compared between the ones whose targeted biopsy indicated PCa, while their systematic biopsy results showed benign lesions, and the ones with positive systematic biopsy for PCa, using student *t*‐test. Moreover, cancer cases were divided into two groups based on PSA equal or more than 10, or below it. The pathology results were compared between these two groups and the abovementioned pathologic subgroups using the chi‐square test. IBM SPSS Statistics 22 (SPSS Inc., Chicago, IL, USA) was used for statistical analysis. A *p*‐value < 0.05 was considered statistically significant.

## 3 Results

**FIGURE 1:**
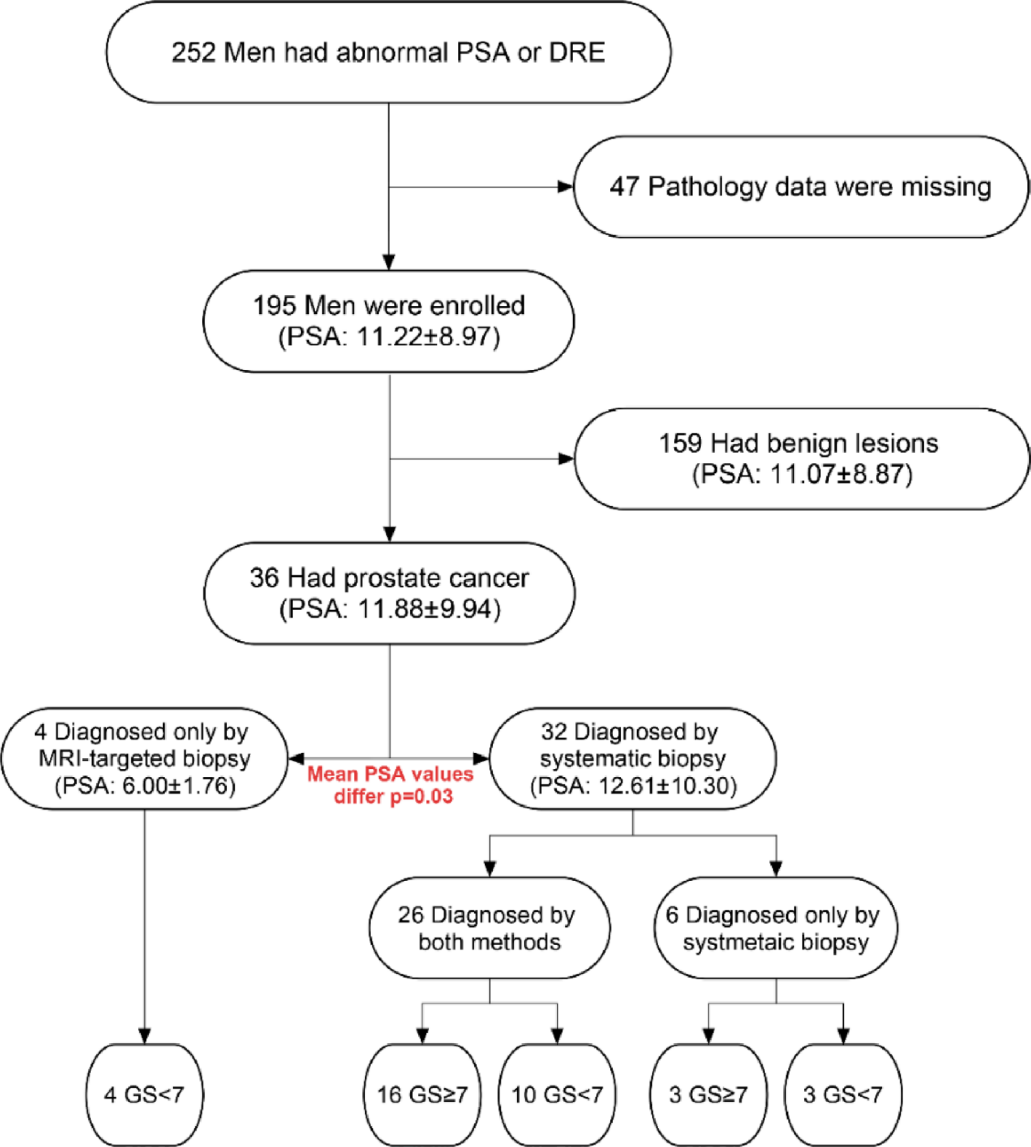
Flowchart of the study population.

**FIGURE 2:**
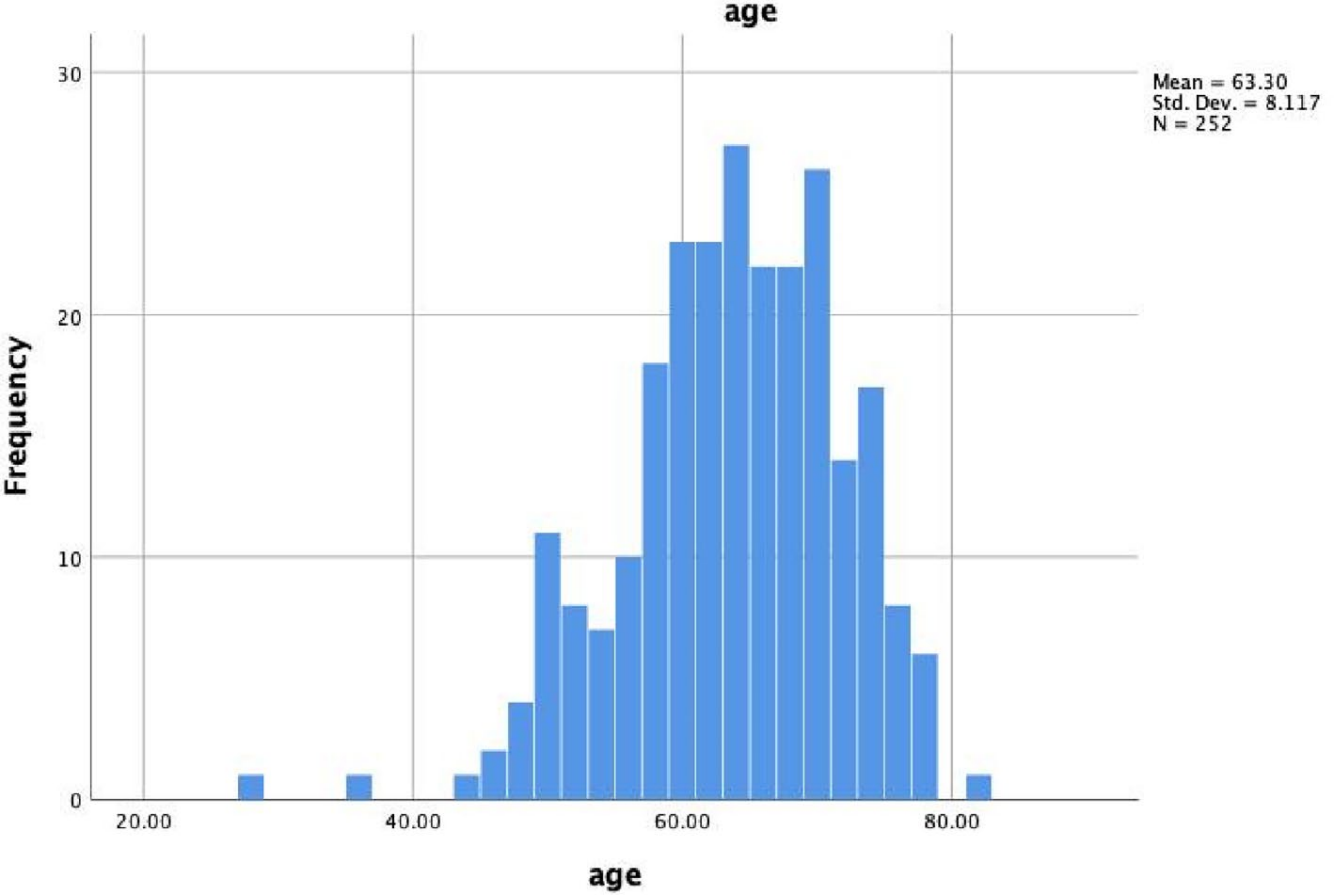
The age of all men examined in the study. The range of their age was from 28 to 82 years old. Its upper quartile was 58.25 and its lower quartile was 69.75.

The flowchart in Figure 1 summarizes the results. Data from 252 men with PSA ≥ 3 ng/mL or abnormal DRE, and a negative history of PCa were available in our database. Among 252 men, the average age was 63.30 years with a standard deviation of 8.11 years (Figure 2).

Pathology results for 47 men were missing, so 195 men with a mean PSA of 11.22 ± 8.97 ng/mL were enrolled, all of whom underwent mpMRI and combined targeted and systematic prostate biopsy. Of these, 81 (41.53%) had a history of smoking, 49 (25.12%) had a history of alcohol consumption, 13 (6.6%) had a history of cardiovascular diseases and 35 (17.94%) patients had a history of diabetes. Furthermore, our findings indicated that 56 (28.71%) patients had a positive family history of PCa in their brother, father, or grandfather.

Digital Rectal Exam (DRE) performed among 195 patients showed mild, moderate, and huge enlargement in 13 (6.6%), 114 (58.46%), and 32 (16.41%) patients, respectively. Reports of 36 (18.46%) men were missing.

Lesion‐based cancer detection rates (CDRs) of prostate cancer for (Prostate Imaging–Reporting and Data System) (PI‐RADS) 1, 2, 3, 4, and 5 lesions were 0%, 10 (5.12%), 64 (32.82%), 70 (35.89%), and 20 (1024%), respectively. The information for 31 (15.89%) patients was not available.

The association between patient characteristics and pathology results is summarized in Tables 1 and 2. Moreover, among the 36 cases of prostate cancer, 17 (47.22%) had a Gleason score < 7, 11 (30.55%) had a Gleason score of 7, and 8 (22.22%) had a Gleason score > 7.

**TABLE 1:** The association between patient characteristics and pathology results.

Variable		*N*	Age (year) [Mean (SD)]	Smoking		Alcohol		Family history of cancer		Diabetes mellitus		IHD		BMI (kg/m^2^) [Mean (SD)]
				Yes (%)	No (%)	Yes (%)	No (%)	Positive (%)	Negative (%)	Positive (%)	Negative (%)	Positive (%)	Negative (%)	
The pathology	Cancer	36	63.61 (6.55)	19 (52.8%)	17 (47.2%)	12 (33.3%)	24 (66.4%)	4 (11.11%)	32 (88.9%)	4 (11.1%)	32 (88.9%)	3 (8.3%)	33 (91.7%)	26.64 (3.24)
	Benign	159	62.32 (8.13)	62 (39.0%)	97 (61.0%)	37 (23.3%)	122 (76.7%)	52 (32.7%)	107 (97.3%)	31 (19.5%)	128 (80.5%)	10 (6.3%)	149 (93.7%)	26.76 (9.81)
Total		195	62.55 (7.86)	81 (41.5%)	114 (58.5%)	49 (25.1%)	146 (74.9%)	56 (28.7%)	139 (71.3%)	35 (17.9%)	160 (82.1%)	13 (6.7%)	182 (93.3%)	26.74 (7.96)
*p‐*value^a^, ^b^			0.313	0.130		0.209		**0.010**		0.236		0.657		0.893

*Note:* A *p*‐value of < 0.05 was considered statistically significant.

Abbreviation: BMI, body mass index.

^a^ Independent *t*‐test.

^b^ Chi‐squared test.

**TABLE 2:** The association between PIRADS and pathology results.

					PIRADS		
Variable		*N*	1	2	3	4	5
			*N* (%)	*N* (%)	*N* (%)	*N* (%)	*N* (%)
The pathology	Cancer	36	0 (0%)	9 (6.7%)	59 (44.00%)	58 (43.3%)	8 (6.0%)
	Benign	159	0 (0%)	1 (3.3%)	5 (16.7%)	12 (40.0%)	12 (40%)
Total		195	0 (0%)	10 (6.1%)	64 (39.0%)	70 (42.7%)	20 (12.2%)
*p‐*value^a^			0.000				

^a^ Chi‐squared test.

Biopsy results indicated benign lesions in 159 men (PSA: 11.07 ± 8.87 ng/mL), while PCa was found in 36 men (PSA: 11.88 ± 9.94 ng/mL). Systematic biopsy was able to detect cancer in 32 patients, while missing 4 PCa cases, which were only detected by targeted biopsy. Mean PSA level was statistically different between these two groups (PSA: 12.61 ± 10.30 and 6.00 ± 1.76, respectively; *p* = 0.03).

Seventeen men had a Gleason score < 7, indicating clinically insignificant PCa. All of the cases detected solely by targeted biopsy belonged to this group. Moreover, these 4 men all had PSA levels below 10. Also, in PSA ≤ 10 ng/mL, six cases were recognized only by systematic biopsy. No PCa was missed by either of the two methods in men with PSA > 10 ng/mL (Table 3).

**TABLE 3:** The categorizing PSA among patients with prostate cancer.

Prostate cancer pathology		PSA level	
		≤ 10	> 10
Diagnosed by	Targeted biopsy only	4	0
	Both methods	13	13
	Systematic biopsy only	6	0
*p*‐value		**0.02**	

*Note:* A *p*‐value of < 0.05 was considered statistically significant.

## 4 Discussion

In this study, out of 195 men, 36 were diagnosed with PCa. PCa was diagnosed by both MRI‐TBx and TRUS‐Bx in 26 men, half of whom had PSA > 10 ng/mL. At PSA ≤ 10 ng/mL, PCa would have been missed in 4 men (11.1%) had MRI‐TBx not been done, and in 6 men (16.6%) had TRUS‐Bx not been done.

Organized screening programs by means of testing PSA level and subsequent performance of systematic TRUS‐Bx in men with elevated PSA levels reduces PCa mortality [10]. However, no net benefit is suggested for screening, mainly due to high rates of over diagnosis. A large proportion of men will undergo radical treatment and suffer permanent complications, including incontinence and impotence [11]. Besides, prostate biopsy—regardless of the method—is an unpleasant procedure, typically accompanied by usually‐self‐limiting complications, including hematuria, hematospermia, rectal bleeding, urinary tract infection, erectile dysfunction, and infections [12]. Therefore, studies have been conducted in an effort to reduce the number of men undergoing prostate biopsy and the number of samples taken during each procedure. These issues were primarily addressed by assessing the value of each biopsy method in detection of PCa.

The PRECISION (Prostate Evaluation for Clinically Important Disease: Sampling Using Image Guidance or Not?) group conducted a randomized trial with 500 biopsy‐naïve men, who were assigned to either systematic TRUS‐Bx without mpMRI, or mpMRI followed by MRI‐TBx for those showing PI‐RADS ≥ 3 lesions. Detection of csPCa was significantly higher in the second group (26% vs. 38%, adjusted difference, 12 percentage points; 95% confidence interval [CI] 4–20; *p* = 0.005). They did not, however, examine whether combining the two methods would result in higher csPCa detection [5]. The MRI‐FIRST trial analyzed 251 men and found csPCa in 94 of them. Cancer was detected in 62 men by both methods, in 13 men by systematic TRUS‐Bx only, and in 19 men by MRI‐TBx only. MRI‐TBx was slightly superior in detection of csPCa, but not significantly (32.3%, 95% CI 26.5–38.4 vs. 29.9%, 95% CI 24.3–36.0; *p* = 0.38). They concluded that, despite no difference between the two methods in the detection of csPCa, combining both methods seems necessary, as it provides substantial added value in biopsy‐naïve men [13]. In the GÖTEBORG‐2 trial, 17 980 men underwent prostate MRI, and subsequently, were randomly assigned to either systematic plus targeted biopsy, or targeted biopsy alone. Clinically‐insignificant PCa was detected at half the rate in the latter group (1.2% vs. 0.6%, −0.7 percentage points difference, 95% CI −1.0 to −0.4; Relative Risk [RR] 0.46, 95% CI 0.33–0.64; *p* < 0.001). The RR of csPCa detection in the MRI‐TBx‐only group, compared to the combined methods group, was 0.81 (95% CI 0.60–1.1). In the combined group, 10 csPCa cases were diagnosed only by systematic TRUS‐Bx. They concluded that omitting systematic biopsy for early detection of PCa in men with elevated PSA levels reduces overdiagnosis by half, to the detriment of delayed detection of intermediate‐risk cancers in a small percentage of cases [14].

Considering the results of the mentioned studies, it seems inevitable to perform both MRI targeted, and systematic biopsy in a clinically‐suspicious man. In fact, Dell'Oglio et al. were so adamant about not omitting one biopsy method, that they titled their article “There Is No Way to Avoid Systematic Prostate Biopsies in Addition to Multiparametric Magnetic Resonance Imaging Targeted Biopsies” [9]. They tried to develop an individualized risk calculator by identifying independent predictors of presence of csPCa outside the MRI‐detected lesions. The aim was to exclude men with a calculated low probability of harboring csPCa from undergoing systematic biopsy. However, since the number of spared systematic biopsies at the expense of missing csPCa was negligible, they recommended against MRI‐TBx‐only approach.

While the recent studies are mainly focused on the role of MRI in finding suspicious prostate lesions and guiding biopsy, and even further, on proposing to replace the conventional systematic TRUS‐Bx with MRI‐TBx, the limitations must be considered. Prostate MRI and trained professionals to interpret its findings are not accessible everywhere. Even if we consider tele‐medicine as an answer to the interpretation problem, the financial burden still remains. As Kim et al. reported, 1–1.2 million prostate biopsies are performed in United States each year. Performing MRI before each biopsy would cost a total of $3 billion, approximately 15% of the total cost of managing PCa [15]. With these in mind, Wang et al. questioned the PSA threshold at which MRI‐TBx should be added to systematic biopsy. They found that in PSA > 4.3 ng/mL, adding MRI‐TBx significantly increases csPCa detection (*p* = 0.031) [16]. In the study of Byun et al. MRI‐TBx significantly detected a higher rate of csPCa at PSA levels 4–10 ng/mL (35.0% vs. 26.6%, *p* = 0.033). However, detection rate did not differ significantly between two methods at PSA < 4 (12.0% vs. 16.0%, *p* = 0.342) and PSA > 10 (78.0% vs. 80.0%, *p* = 0.596) [17]. These findings emphasize the importance of performing both methods at PSA levels 4–10. Our results indicate that at PSA > 10, either of the methods yields the same diagnosis. However, at PSA ≤ 10 discrepancies are witnessed. TRUS‐Bx was able to diagnose two clinically‐significant and four clinically‐insignificant PCas, while MRI‐TBx was negative. MRI‐TBx diagnosed four clinically‐insignificant PCas in the absence of a positive TRUS‐Bx. Even‐though the cases diagnosed only by MRI‐TBx all had a Gleason score < 7, this might be due to the study's sample size.

The cost‐effectiveness of performing MRI prior to prostate biopsy has been assessed by several other studies. They suggest comparable costs and quality of life, in favor of performing pre‐biopsy MRI. This may root in fewer insignificant cancer diagnosis, earlier significant cancer detection, and fewer biopsy repetitions [5, 18, 19, 20]. So, it remains unclear and yet an open area of discussion on how to proceed. Considering our results, we propose to apply the combination of systematic TRUS‐Bx and MRI‐TBx in the assessment of men with PSA ≤ 10 ng/mL and one or more PI‐RADS ≥ 3 lesion(s) on pre‐biopsy mpMRI. In other terms, the attempts to perform only one method of biopsy, are better to be preserved for men with PSA > 10.

Our study has some limitations. It is a retrospective study done at a single institution. Although it is a tertiary referral center, this raises concerns about selection bias. One limitation of our study was the missing data on some variables including PSA density, PSA‐ velocity, and free/total PSA. Reporting of the prostate MRIs and MRI‐targeted biopsies are done by a single experienced radiologist and urologist, respectively. This is a strength of our study, as it reduces the heterogeneity between the obtained results. Radiologists and urologists with less expertise, especially outside an expert center, may not achieve the same diagnostic yield. Regarding the learning curve, it would be beneficial for training programs to incorporate high volumes of prostate MRI reporting under the supervision of expert radiologists, as proposed by Kasivisvanathan et al. [5].

## 5 Conclusion

Despite attempts to perform only one biopsy method in men with clinical suspicion of prostate cancer, we propose that at least in men with PSA ≤ 10 ng/mL, both systematic and MRI‐targeted biopsies be performed.

## Author Contributions

**Solmaz Ohadian Moghadam:** conceptualization and design of the study (equal), funding acquisition (equal), investigation (equal), methodology (equal), supervision (equal), collecting and analyzing and interpretation of the data (equal), drafting and critical revision (lead). **Mohammad Haddadi:** methodology (equal), investigation (equal), drafting (equal). **Erfan Amini:** conceptualization and design of the study (lead), collecting and analyzing and interpretation of the data (equal), formal analysis (equal), drafting and revision (equal). **Seyed Ali Momeni:** collecting and analyzing and interpretation of the data (equal), drafting and revision (equal). **Masoud Bitaraf:** methodology (equal), supervision (equal), analyzing and interpretation of the data (equal), drafting and revision (equal). **Mohammad Reza Nowroozi:** conceptualization and design of the study (equal), supervision (lead), analyzing and interpretation of the data (equal), drafting and revision (equal).

## Ethics Statement

This study is approved by research ethics committee of IKHC (IR.TUMS.IKHC.REC.1399.528) and all methods were carried out in accordance with the declaration of Helsinki.

## Conflicts of Interest

The authors declare no conflicts of interest.

## Data Availability

The data that support the findings of this study are available from the corresponding author upon reasonable request.
